# Guselkumab-Associated Pulmonary Disease and Diffuse Alveolar Hemorrhage with Drug Rash with Eosinophilia and Systemic Symptoms

**DOI:** 10.7759/cureus.34623

**Published:** 2023-02-04

**Authors:** Khizar Hamid, Marlee E Jones, Jiannan Huang, John C Yu

**Affiliations:** 1 Internal Medicine, University of South Dakota Sanford School of Medicine, Sioux Falls, USA; 2 Pulmonary and Critical Care Medicine, Sanford University of South Dakota (USD) Medical Center, Sioux Falls, USA

**Keywords:** acute respiratory distress syndrome (ards), disease-modifying antirheumatic drugs (dmards), diffuse alveolar hemorrhage, drug reaction with eosinophilia and systemic symptoms (dress), idiopathic interstitial pneumonia

## Abstract

Psoriasis is a common skin condition worldwide. Moderate-to-severe disease is treated with biologic or non-biologic disease-modifying anti-rheumatic drugs. These include tumor necrosis factor (TNF)-a inhibitors, interleukin (IL)-17 inhibitors, and IL-23 inhibitors. Case reports of inhibitors of TNF-a and IL-12p40 subunits causing interstitial pneumonia (IP) have been published in the literature, but no case of anti-IL-23p19 subunit biologics causing IP and acute respiratory distress syndrome (ARDS) has been reported before. We report a case of a patient with restrictive lung disease secondary to a body mass index of 36.54 kg/m^2^, obstructive sleep apnea, and psoriasis, who developed IP and ARDS presumed to be secondary to guselkumab, an anti-IL-23p19 subunit monoclonal antibody. He was on ustekinumab, an anti-IL-12/23p40 for the treatment of psoriasis, but was switched to guselkumab eight months before the presentation, and since then he had been complaining of progressive shortness of breath. He initially presented to the hospital after having drug reaction with eosinophilia and systemic symptoms (DRESS) after being started on amoxicillin for a tooth infection. He was treated with high-dose intravenous steroids but developed progressive shortness of breath. Broad-spectrum antibiotics were added. An extensive infectious, autoimmune, and hypersensitivity work-up was undertaken, which returned negative. A bronchoscopy with bronchoalveolar lavage was performed, which revealed diffuse alveolar hemorrhage (DAH). His lung imaging and oxygenation progressively got worse; hence, no lung biopsy was taken. He was intubated and required inhaled nitric oxide, but due to the lack of improvement, the family elected for comfort measures, and the patient was extubated and passed away. To our knowledge, this is the first case of an association between guselkumab, IP, ARDS, and DAH. Rare instances of DAH with DRESS have been reported before. Whether it was DRESS or guselkumab that caused DAH was uncertain in our patient. Clinicians should monitor for DAH and shortness of breath in patients on guselkumab so that more data can be obtained and studied in the future.

## Introduction

Psoriasis is a common skin disease occurring worldwide [1]. In patients with moderate-to-severe disease, treatment with biologic or non-biologic disease-modifying anti-rheumatic drugs (DMARDS) can result in improvement of joint and skin symptoms along with the prevention of permanent structural damage [2]. Biologic agents used for the treatment of psoriasis are divided into three groups, namely, tumor necrosis factor (TNF)-a inhibitors, interleukin (IL)-17 inhibitors, and IL-23 inhibitors [3]. Cases of TNF-a inhibitors such as infliximab and adalimumab causing interstitial pneumonia (IP) have been reported in the literature [4,5]. Ustekinumab, which acts on the IL-12/23p40 subunit, has also been reported to cause IP and even acute respiratory distress syndrome (ARDS) [6,7]. No case of guselkumab, which is an anti-IL-23p19 subunit monoclonal antibody, causing IP or ARDS has been reported in the literature before [2].

## Case presentation

A 57-year-old man presented to the emergency department with complaints of generalized pruritic bullous rash involving the oral mucosa along with swelling of his face and legs. His past medical history was notable for obstructive sleep apnea (OSA) treated with continuous positive airway pressure with 2 L/min of nocturnal oxygen, restrictive lung disease (RLD) secondary to class III obesity with a body mass index (BMI) of 36.54 kg/m^2^, and psoriasis on guselkumab. He had been switched from ustekinumab to guselkumab eight months before and was complaining of progressive shortness of breath since then. Two weeks before admission, he was started on amoxicillin for a tooth infection. On the fourth day of treatment, he developed an erythematous maculopapular rash that rapidly became bullous. It started on his back and progressed to his chest, abdomen, and face, eventually involving all four limbs. Oral steroids were started, but he developed swelling in his face and legs and hence discontinued taking them. The swelling got progressively worse, and he developed oral sores, prompting him to come to the hospital. At presentation, his labs were notable for an eosinophil count of 33% of 25.5 K/uL white blood cells (Table 1).

**Table 1:** Labs notable for an eosinophil count of 33% of 25.5 K/uL white blood cells.

Lab	Value	Reference Range
White blood cells	25.5 K/uL	4.5–11.0 K/uL
Hemoglobin	16.5 g/dL	14.0–17.5 g/dL
Platelet	226 K/uL	140–450 K/uL
Sodium	134 mmol/L	133–145 mmol/L
Potassium	3.7 mmol/L	3.5–5.1 mmol/L
Chloride	99 mmol/L	98–109 mmol/L
Bicarbonate	24 mmol/L	21–31 mmol/L
Blood urea nitrogen	18 mg/dL	7–25 mg/dL
Creatinine	1.1 mg/dL	0.7–.3 mg/dL
Aspartate aminotransferase	19 U/L	13-39 U/L
Alanine transaminase	22 U/L	4–33 U/L
Alkaline phosphatase	60 U/L	34–104 U/L

**Figure 1:**
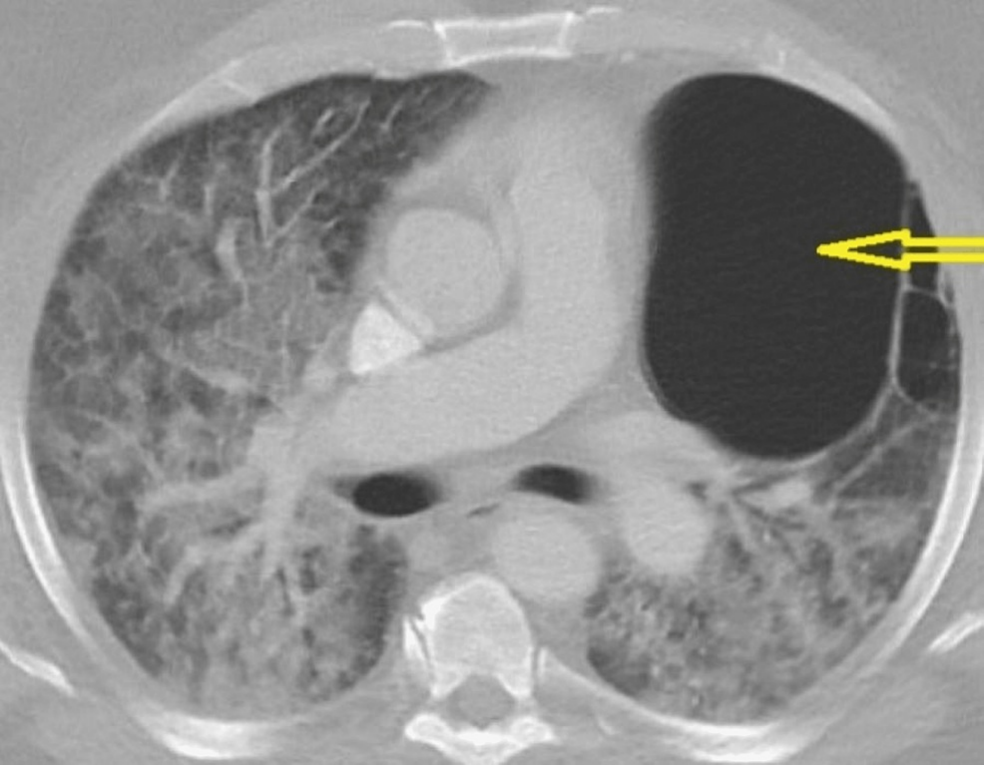
Computed tomographic angiogram. No pulmonary embolism was noted. A 13-cm bulla was noted in the left lung (yellow arrow) with bilaterally ground-glass consolidation. Differential diagnosis includes pulmonary edema, pneumonia, or various causes of alveolitis.

Concern for drug rash with eosinophilia and systemic symptoms (DRESS) and toxic epidermal necrolysis (TEN) led to a left upper back skin biopsy, which revealed epidermal spongiosis with mild interface alteration, perivascular lymphoid infiltrate, and many eosinophils. DRESS could not be ruled out, however; TEN was excluded, and he was started on intravenous (IV) methylprednisolone at 125 mg/day. On day 2 of hospitalization, he developed a cough with bloody sputum production and progressive shortness of breath requiring bilevel positive airway pressure with a FiO_2_ of 60% alternating with humidified heated high flow (HHF) at 40 L/min. A computed tomographic angiogram (CTA) of his chest revealed a 13-cm bulla in the left lung with bilateral ground-glass consolidation (Figure 1).

Further review revealed he had worked on tires that involved grinding and exposure to asbestos and rubber dust. He denied recent travel, smoking, owning pets, or having any exposure to sick contacts. IV meropenem 500 mg every 12 hours for five days and IV levofloxacin 750 mg daily for nine days were added to systemic steroids. Extensive infectious work-up including comprehensive respiratory panel, legionella and streptococcal pneumonia urine antigens, methicillin-resistant *Staphylococcus aureus* PCR, HIV, SARS-CoV-2, and mycoplasma immunoglobulin M were negative. Sputum culture grew *Rothia mucilaginosa*, which was a contaminant, while blood cultures returned negative. Fungal testing for aspergillus, blastomyces, coccidioides, histoplasma, *Pneumocystis jirovecii* (PCJ), and beta-D-glucan returned negative. Extensive autoimmune testing was also unremarkable (Table 2).

**Table 2:** Extensive autoimmune testing negative. ANA, antinuclear antibody; ANCA, antineutrophil cytoplasmic antibodies; dsDNA, double-stranded DNA; CCP, cyclic citrullinated peptide; Sm, anti-Smith; SS-A/Ro, Sjögren's syndrome antigen A; SS-B/La, Sjögren's syndrome antigen B; Jo-1, histidyl-tRNA synthetase; RNP, ribonucleoprotein; Scl-70, scleroderma antigen 70; IgG, immunoglolbulin G; Ab, antibody

Lab	Value	Reference Range
ANA screen	Negative	Negative
ANCA screen	Negative	Negative
Anti-dsDNA (0.0–29.9 IU)	Negative	<12.3 IU
Glomerular basement membrane	Negative	Negative
C3 complement	126 mg/dL	82–185 mg/dL
C4 complement	17.3 mg/dL	15.0–53.0 mg/dL
CCP antibodies	0.9 U/mL	0.0–6.9 U/mL
Myeloperoxidase antibody	Negative	Negative
Proteinase 3 antibody	Negative	Negative
Rheumatoid factor	< 13 IU/mL	0–29 IU/mL
SM antibody	Negative	Negative
SS-A/Ro Ab, IgG	Negative	Negative
SS-B/La Ab, IgG	Negative	Negative
Jo 1 Ab, IgG	Negative	Negative
RNP Ab, IgG	Negative	Negative
Scl 70 Ab, IgG	Negative	Negative

**Figure 2:**
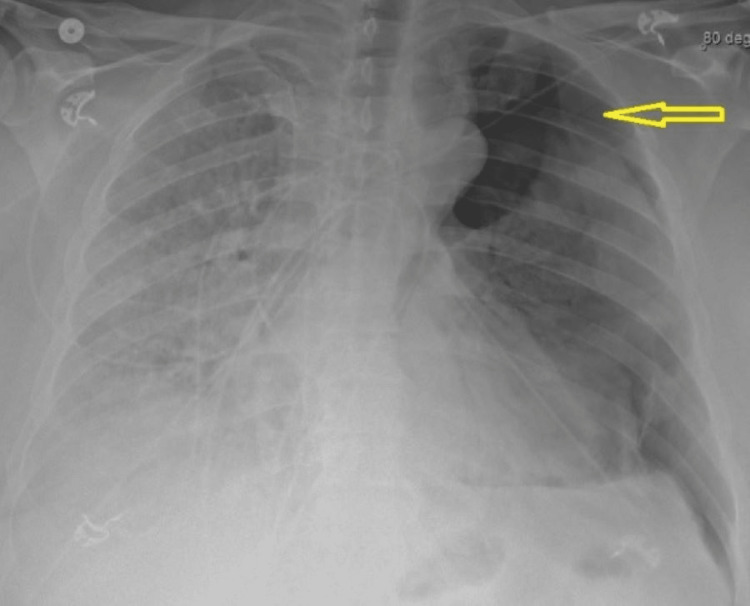
Chest X-ray. Yellow arrow points toward the apical pneumothorax. Considerable opacification of the lung fields was also noted.

His QuantiFERON testing returned as indeterminate, but he denied any exposure to tuberculosis. herpes simplex virus-1 DNA testing returned positive, and acyclovir was added. His oxygen requirements improved to 4-5 L/min of oxygen via nasal cannula, and bronchoscopy with bronchoalveolar lavage (BAL) was performed, which revealed diffuse alveolar hemorrhage (DAH). The methylprednisolone dose was increased to 1 g/day. BAL fungal testing, PCJ PCR, and BAL mycobacterial, fungal, and bacterial cultures also returned negative. No eosinophils were noted in BAL, and the hypersensitivity pneumonitis panel returned negative. Following BAL, his oxygen requirements increased to 100% FiO_2_ with 50 L/min of oxygen via HHF, leading to concern for venous thromboembolism. CTA was not obtained due to ongoing acute kidney injury (AKI); a ventilation-perfusion scan was not obtained due to abnormalities noted on chest imaging. Bilateral upper and lower limb duplex venous ultrasound returned negative for deep venous thrombosis. The next day, he developed severe respiratory distress requiring maximum noninvasive ventilatory support. Chest X-ray showed a left loculated apical pneumothorax. The patient was intubated and sedated, and a chest tube was inserted (Figure 2).

**Figure 3:**
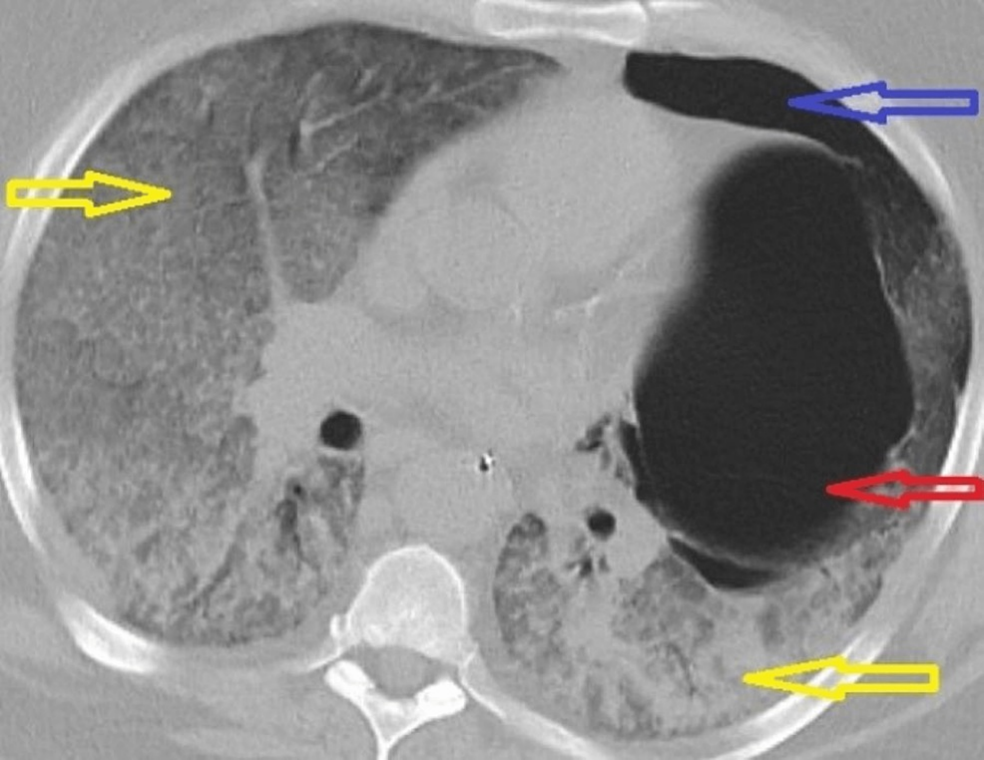
Computed tomography of the chest without contrast. Diffuse bilateral airspace consolidation (yellow arrows), existing pulmonary bleb (red arrow), and unresolved pneumothorax (blue arrow) were noted.

His AKI worsened, and continuous renal replacement therapy was started. Methylprednisolone was tapered to 125 mg IV every 6 hours for three days followed by 60 mg IV every 6 hours. Atovaquone was added for PCJ prophylaxis, and he was paralyzed with cistacurorium and initiated on inhaled nitric oxide (iNO) for refractory hypoxemia. Lung biopsy was not obtained due to patient instability, and an attempt to wean him off iNO resulted in recalcitrant desaturation. Repeat computed tomography of the chest revealed progression of bilateral infiltrates with little healthy lung tissue remaining (Figure 3).

He was not a candidate for salvage lung transplantation due to his BMI, continued paralysis, and need for iNO. Since he was unable to participate in any decision-making, a palliative care meeting was held, and the family elected to pursue compassionate extubation.

## Discussion

Biologics used for the treatment of psoriasis can cause IP and ARDS [4,6,7]. ARDS is defined according to the Berlin criteria based on the ratio of the partial pressure of oxygen in the arterial circulation to the partial pressure of oxygen in the alveoli [8]. Interstitial lung disease is an umbrella term and includes more than 200 different diseases displaying considerable variation. They can be broadly classified into those with an identifiable cause and those without [9]. The current gold standard for diagnosis is the multidisciplinary panel discussion and determination of the need for surgical lung biopsy for histopathological assessment. There has been growing interest in transbronchial lung biopsy as well for diagnosis [10]. Our patient was medically unstable; hence, he did not undergo a lung biopsy. Despite an extensive infectious, autoimmune, and hypersensitivity work-up, no secondary cause of the patient's pulmonary findings was identified. He was initially on ustekinumab, which can cause pulmonary injury, which is reversible upon discontinuation and treatment with steroids; hence, it was not considered the cause of his pulmonary findings as he had not been on it for eight months before presentation [7]. Due to his extensive negative work-up, it was thought that guselkumab could have been the cause of his pulmonary findings, especially since he had been complaining of progressive shortness of breath since the beginning of the switch. The patient already had poor pulmonary reserve due to his RLD and OSA. It is possible that the inflammatory challenge from his allergic reaction to amoxicillin accelerated the pulmonary effects of guselkumab. More research is needed, and clinicians should monitor for the development of pulmonary toxicity in patients taking guselkumab or other related biologics, especially in patients who are prone to allergic reactions. Guselkumab is also not currently known to be associated with DAH, which was noted in our patient. Rare instances of DRESS causing DAH have been reported before; hence, it is difficult to determine if it was DRESS or guselkumab that was the cause of DAH [11]. No doubt, DAH represents a syndrome of severe lung injury of varying etiologies that are associated with microvascular damage resulting in intra-alveolar red blood cell accumulation [12]. Awareness of this possibility can help better define this association in the future since currently guselkumab is considered a drug with relatively few adverse effects [13].

## Conclusions

Cases of TNF-a inhibitors and ustekinumab causing IP and ARDS have been reported. No case of guselkumab causing IP and ARDS has been reported before. Having preexisting RLD, OSA, psoriasis, propensity for allergic reactions, and prior treatment with ustekinumab might make guselkumab’s pulmonary effects worse. DRESS can also present with DAH and may be associated with guselkumab. If patients have been transitioned to guselkumab, especially from ustekinumab, and complain of shortness of breath, guselkumab as a cause should be considered.
